# Meta-analysis of spleen-strengthening and phlegm-resolving herbal therapy for airway mucus hypersecretion in chronic obstructive pulmonary disease

**DOI:** 10.3389/fmed.2026.1776303

**Published:** 2026-03-19

**Authors:** Mengyao Chen, Jiamin Lu, Yu Wei

**Affiliations:** Jiangsu Province Hospital of Chinese Medicine, Affiliated Hospital of Nanjing University of Chinese Medicine, Nanjing, Jiangsu, China

**Keywords:** chronic obstructive pulmonary disease, meta-analysis, MUC5AC, spleen-strengthening and phlegm-resolving, traditional Chinese medicine

## Abstract

**Objective:**

To systematically evaluate the clinical efficacy and safety of Chinese herbal medicine based on spleen-strengthening and phlegm-resolving (SSPR) therapy for airway mucus hypersecretion in chronic obstructive pulmonary disease (COPD) from an evidence-based medicine perspective, and to provide a scientific basis for TCM diagnosis and treatment of COPD-related mucus hypersecretion.

**Methods:**

This study was conducted following PRISMA (Preferred Reporting Items for Systematic Reviews and Meta-Analyses) guidelines. We systematically searched CNKI, Wanfang Data, VIP, CBM, PubMed, Embase, and Cochrane Library for clinical studies published between January 1, 2010, and September 1, 2025, on SSPR-based herbal interventions for COPD mucus hypersecretion. Two researchers independently screened the studies according to predefined inclusion/exclusion criteria, extracted data, performed cross-checking, and assessed study quality. Meta-analysis was performed using RevMan 5.4, with publication bias evaluated by Egger's test in Stata 18.

**Results:**

Sixteen studies involving 1,468 COPD patients with mucus hypersecretion were included. Meta-analysis showed that for acute exacerbation of COPD: SSPR combined with conventional Western therapy significantly improved Treatment effectiveness rate (*P* < 0.0001), FVC (*P* = 0.02), and absolute FEV_1_ (*P* < 0.00001). SSPR also significantly reduced MUC5AC (*P* = 0.001), TCM syndrome scores (*P* < 0.00001), and IL-8 (*P* < 0.00001) compared to Western therapy alone. No significant difference in FEV_1_/FVC ratio improvement (*P* = 0.22) was observed. For stable COPD: SSPR combination therapy significantly improved Treatment effectiveness rate (*P* < 0.0001), FVC (*P* < 0.00001), absolute FEV_1_ (*P* < 0.00001), and FEV_1_/FVC ratio (*P* = 0.001). SSPR also significantly reduced TCM syndrome scores (*P* = 0.0004) and IL-8 (*P* < 0.00001). No significant difference in MUC5AC reduction (*P* = 0.23) was observed. Three studies reported no adverse events, while two showed no statistically significant difference in adverse event rates between groups.

**Conclusion:**

SSPR herbal therapy combined with conventional Western treatment demonstrates superior clinical efficacy for managing airway mucus hypersecretion in both acute exacerbation and stable COPD compared to Western therapy alone, with comparable safety profiles. However, the included studies were generally of low methodological quality, highlighting the need for larger, higher-quality RCTs for further validation.

**Systematic Review Registration:**

The protocol was registered on PROSPERO (CRD420251171076).

## 1 Introduction

Chronic obstructive pulmonary disease (COPD) is a heterogeneous pulmonary disorder characterized by persistent and typically progressive airflow limitation caused by chronic respiratory symptoms (dyspnea, cough, sputum production, and/or exacerbations) resulting from airway abnormalities (bronchitis, bronchiolitis) and/or alveolar damage (emphysema) (1). Currently, COPD affects more than 400 million people worldwide (2), with a prevalence exceeding 13% among individuals aged 40 years and older (3). With increasing smoking prevalence in low-income countries and population aging in high-income nations, COPD prevalence is projected to continue rising (4). Global estimates suggest a 23% increase in COPD cases among adults ≥ 25 years from 2020 to 2050, reaching nearly 600 million patients by 2050 (5). The Global Initiative for Chronic Obstructive Lung Disease classifies COPD into acute exacerbation and stable phases based on clinical manifestations (1).

AMH represents a fundamental pathophysiological feature and clinical hallmark of COPD (6). The characteristic symptoms of chronic cough and sputum production demonstrate strong associations with adverse clinical outcomes, including reduced exercise capacity, frequent acute exacerbations and increased mortality risk (7). During stable phases, pathological goblet cell hyperplasia coupled with impaired mucociliary clearance creates favorable conditions for microbial colonization and recurrent infections (8). In acute exacerbations, excessive mucus secretion exacerbates airway obstruction, leading to rapid pulmonary function decline and increased risks of clinical deterioration. The mucus consists primarily of MUC5AC mucins, which are abnormally upregulated in COPD patients, contributing to airway obstruction and disease progression (9). These findings underscore the clinical significance of managing AMH in COPD.

Modern medical research has revealed that AMH is a core pathological feature of COPD (1). Its pathogenesis involves multiple aspects: cigarette smoke and other stimuli lead to goblet cell metaplasia and increased mucus secretion (10); inflammatory mediators such as IL-8 and IL-13, and neutrophil elastase activate signaling pathways including epidermal growth factor receptor (EGFR) to promote mucin synthesis (11, 12); pathogens directly or indirectly damage airways, causing airway inflammation, increased secretions, and mucociliary dysfunction, thereby impairing mucus clearance (13). These pathological changes ultimately result in mucus obstructing airways, airflow limitation, hypoxemia, and increased risk of acute exacerbations. Current conventional therapies for COPD-related mucus hypersecretion primarily involve mucolytics combined with antibiotics or corticosteroids (1). While demonstrating initial efficacy, these treatments are limited by their narrow mechanisms of action, development of drug resistance and significant adverse effects (14).

In contrast, traditional Chinese medicine (TCM) has shown promising therapeutic advantages. In traditional Chinese medicine (TCM) theory, COPD falls under the categories of “lung distention” and “wheezing syndrome” (15). Its basic pathogenesis involves root deficiency and branch excess, where deficiency of lung, spleen, and kidney qi constitutes the root, while phlegm-turbidity, fluid retention, and blood stasis intermingling form the branch. Multiple factors interact and influence each other as both cause and effect, closely related to visceral dysfunction (16). Analyzing the etiology and pathogenesis of COPD AMH: dietary irregularities and fatigue impair spleen transport, leading to internal retention of water-dampness that accumulates into phlegm, which then floods upward to the lungs–this is called “the spleen as the source of phlegm production” (17). Phlegm-turbidity congesting the lungs obstructs qi movement, impairing lung diffusion and descent, further aggravating internal phlegm-dampness–hence “the lungs as the storage vessel for phlegm.” Chronic disease involving the kidneys leads to kidney yang deficiency or constitutional kidney yang insufficiency with life-gate fire decline. This fails to warm and transport spleen yang, worsening water-dampness and perpetuating phlegm production (“fire failing to warm earth”), and impairs the kidney's function of steaming and transforming fluids, allowing cold-water to flood upward as phlegm–thus “the kidneys as the root of phlegm production.” Ultimately, the mutual dysfunction of lung, spleen, and kidney creates a vicious cycle of phlegm-dampness production and retention.

TCM treats COPD AMH through pattern differentiation: for phlegm-heat congesting the lung pattern, clear the lungs and transform phlegm, direct qi downward and calm panting; for phlegm-dampness obstructing the lung pattern, dry dampness and transform phlegm, diffuse and descend lung qi; for phlegm-blood stasis intermingling pattern, strengthen spleen and transform phlegm, quicken blood and dispel stasis; for phlegm clouding the orifices pattern, clear heat and expectorate phlegm, quicken blood and open orifices; for lung-spleen qi deficiency pattern, supplement the lungs and fortify the spleen, direct qi downward and transform phlegm (15). The treatment principle of SSRP directly addresses the spleen deficiency and phlegm-dampness pathogenesis of COPD AMH, aligning with TCM's concept of treating disease at its root. Its efficacy has been validated in numerous clinical studies. However, there is currently a lack of meta-analysis of the effectiveness and safety of SSPR therapy in treating this disease, based on the more recent research evidence available.

This study employs PRISMA (18) guidelines to systematically evaluate randomized controlled trials published since 2010 investigating SSPR therapies for COPD-related mucus hypersecretion. Through meta-analytic integration of research data, we aim to assess the efficacy and safety of these interventions, identify current research gaps and provide direction for future investigations to advance TCM applications in COPD management.

## 2 Materials and methods

### 2.1 Literature search strategy

Database selection: Chinese databases (CNKI, WANFANG DATA, VIP, CBM) and English databases (PubMed, Embase, Cochrane Library).

Search terms:

(COPD[Title/Abstract] OR Chronic Obstructive Pulmonary Disease[Title/Abstract]) AND (mucus[Title/Abstract] OR spleen[Title/Abstract] OR phlegm[Title/Abstract]) AND (Traditional Chinese Medicine[Title/Abstract] OR TCM[Title/Abstract] OR Chinese herbal OR herbal medicine[Title/Abstract]).

Time range: January 1, 2010 to September 1, 2025.

### 2.2 Inclusion and exclusion criteria

#### 2.2.1 Inclusion criteria:

Randomized controlled trials (RCTs);

Participants: COPD patients with phlegm-dampness syndrome diagnosed according to established criteria, such as Guidelines for Diagnosis and Treatment of Chronic Obstructive Pulmonary Disease (19), Global Initiative for Chronic Obstructive Lung Disease (1), Guidelines for TCM Diagnosis and Treatment of Chronic Obstructive Pulmonary Disease (15).

Interventions: Control group: Conventional Western treatment or conventional treatment plus placebo; Experimental group: Conventional treatment plus TCM with SSPR therapy.

Outcome measures: Treatment effectiveness rate; Forced vital capacity (FVC); Absolute value of forced expiratory volume in 1 second (FEV_1_); FEV_1_/FVC ratio; MUC5AC mucin level; TCM syndrome score; Interleukin-8 (IL-8) level.

#### 2.2.2 Exclusion criteria:

Non-RCT studies; non-human studies; unavailable data; duplicate publications; non-journal articles; unpublished studies.

### 2.3 Data extraction and quality assessment

Extracted data included baseline characteristics, interventions, efficacy indicators, and adverse reactions.

Study quality was assessed using the Cochrane Risk of Bias tool, evaluating: Random sequence generation; Allocation concealment; Blinding of participants and personnel; Blinding of outcome assessment; Incomplete outcome data; Selective reporting; Other bias. Two researchers independently assessed each study, with disagreements resolved through discussion or consultation with a third reviewer.

### 2.4 Statistical analysis

Meta-analysis was performed using RevMan 5.4 software. For dichotomous variables, odds ratios (OR) were calculated, while mean differences (MD) or standardized mean differences (SMD) were used for continuous variables, all with 95% confidence intervals (95% CI). Statistical significance was set at *P* < 0.05. Heterogeneity was assessed using *I*^2^ statistics:

*I*^2^ ≤ 50%: fixed-effects model

*I*^2^ > 50%: random-effects model.

Subgroup analyses were conducted based on COPD stages (stable vs. acute exacerbation). Publication bias was evaluated using funnel plots and Egger's test in Stata 18.

## 3 Results

### 3.1 Literature search results

**Figure 1:**
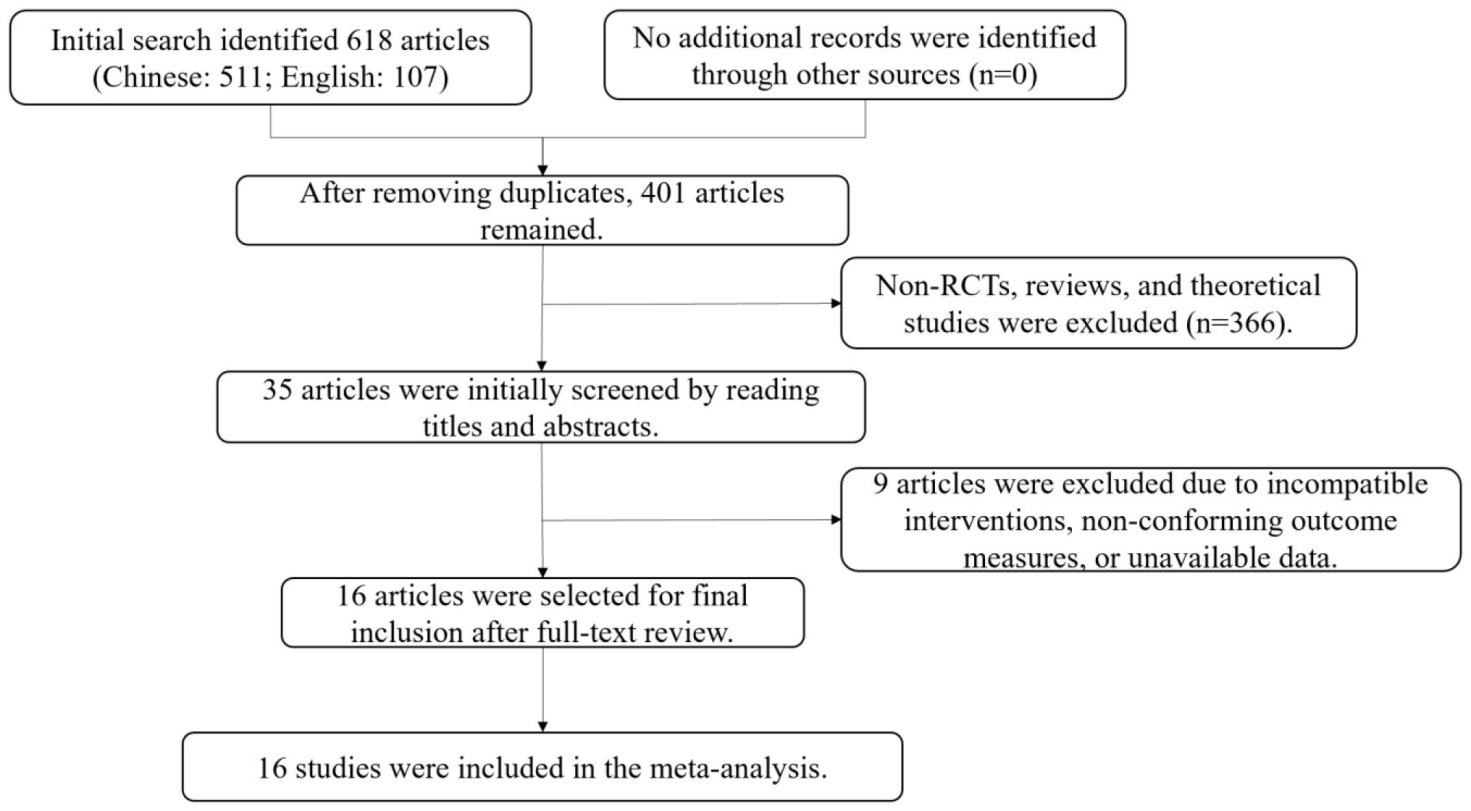
Literature screening flowchart.

Initial searches identified 511 Chinese and 107 English articles (total 618). After removing duplicates, 401 articles remained. After the initial screening by reading titles and abstracts and eliminating non-RCTs, review, theoretical discussion and other ineligible literature, 35 articles remained. Sixteen Chinese RCTs met all inclusion criteria after excluding studies with incompatible interventions, outcomes, or unavailable data. The selection process is shown in Figure 1.

### 3.2 Characteristics of included studies

The meta-analysis included 16 RCTs involving 1,468 COPD patients (intervention group: 739; control group: 729). Sample sizes ranged from 60 to 210 participants. The outcome measures covered: Treatment effectiveness Rate, Forced vital capacity (FVC); Absolute forced expiratory volume in 1 second (FEV_1_); FEV_1_/FVC ratio; MUC5AC mucin level; TCM syndrome score; Interleukin-8 (IL-8) level. Detailed characteristics of the 16 studies are presented in Table 1.

**Table 1:** Characteristics of included studies.

**Study (Author, Year)**	**Sample size**	**Gender (M/F)**		**Intervention**		**Disease stage**	**Outcomes**
		**T**	**C**	**T**	**C**		
Feng et al. (23)	65	16/17	18/14	WM+Qingre Huatan Liqi Compound Formula	WM	AE	⑤⑥
Lin and Liao (24)	70	15/22	13/20	WM+Beimu Gualou San	WM	AE	④⑤⑥
Wu et al. (25)	70	18/17	16/19	WM+Jianpi Bushen Compound Formula	WM	AE	①⑤⑥
Guo et al. (26)	64	19/14	19/12	WM+Qingfei Huatan Decoction	WM	AE	①⑥⑦
Ba et al. (27)	122	33/28	31/30	WM+Jianpi Wenshen Decoction	WM	AE	①②③④⑥⑦
Sun (28)	78	21/18	22/17	WM+Qingjin Huatan Decoction	WM	AE	⑤
Yang et al. (29)	60	25/5	24/6	WM+Tongfei Huatan Decoction	WM	AE	①②③④⑤
Cheng et al. (30)	90	28/17	26/19	WM+Qingjin Baofei Decoction	WM	AE	①⑤⑦
Jiang and Hua (31)	80	24/16	25/15	WM+Yuebi Banxia Decoction	WM	AE	①②③④⑤⑥
Li (32)	90	24/21	26/19	WM+Sanzi Yangqin Decoction (modified)	WM	Stable	①②③⑥⑦
Luo et al. (33)	144	52/20	47/25	WM+Bufei Zhuyu Decoction	WM	Stable	①④⑥
Ma et al. (34)	68	20/15	19/14	WM+Xuanfei Jianpi Fang	WM	Stable	①⑥
Zhang et al. (35)	85	33/10	34/8	WM+Bushen Yifei Fang	WM	Stable	③④?
Liu (36)	98	26/23	29/20	WM+Sanzi Yangqin Decoction (modified)	WM	Stable	①②③⑤⑥⑦
Zhang et al. (37)	210	64/41	67/38	WM+Shenling Baizhu San	WM	Stable	①③⑤
Wang et al. (38)	74	24/13	12/15	WM+Jianpi Bushen Yifei Fang	WM	Stable	②③④⑤

T, Treatment group; C, Control group; WM, Western medicine; AE, Acute exacerbation. Outcome indicators: Treatment effectiveness rate, FVC, Absolute FEV_1_, FEV_1_/FVC ratio, MUC5AC level, TCM syndrome score, ⑦IL-8 level.

### 3.3 Methodological quality assessment

**Figure 2:**
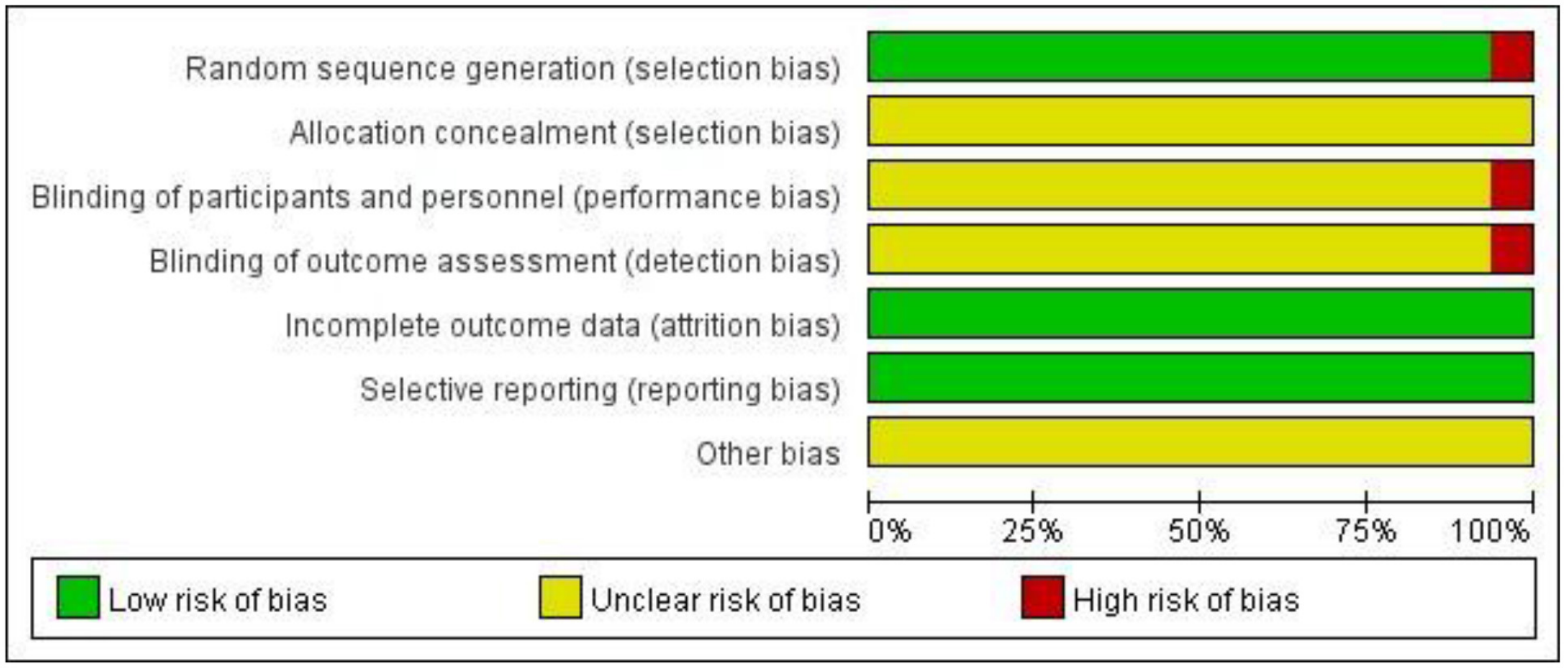
Risk of bias summary.

Among the 16 included studies, 15 reported using randomization for group allocation, with 11 studies specifically employing a random number table method. The remaining 4 randomized studies did not specify the randomization technique despite stating random allocation. Only one study used a non-random sequential enrollment approach based on admission order, which may introduce selection bias. Regarding blinding procedures, the majority of studies failed to report allocation concealment, blinding of participants/personnel, or blinding of outcome assessors. Notably, only one study explicitly stated the absence of blinding for both participants and outcome assessors. All included studies prospectively defined their outcome measures, completed the planned interventions without premature termination, and provided complete outcome reporting. The detailed risk of bias assessment is presented in Figure 2.

### 3.4 The results of the meta-analysis

#### 3.4.1 Treatment effectiveness rate

Treatment effectiveness rate refers to the ratio of the number of patients who have improved after treatment to the total number of patients, which is generally equal to the sum of the cured, markedly effective and effective patients divided by the total number of patients.

Twelve studies included treatment effectiveness rate as an outcome measure. Due to low heterogeneity among studies (*I*^2^ = 0%), a fixed-effects model was used for analysis. The results showed that overall, the addition of Chinese herbal medicine with SSPR effects to conventional Western medicine significantly improved treatment effectiveness rate compared to Western medicine alone [OR = 3.83, 95% CI (2.62, 5.60), *P* < 0.00001].

**Figure 3:**
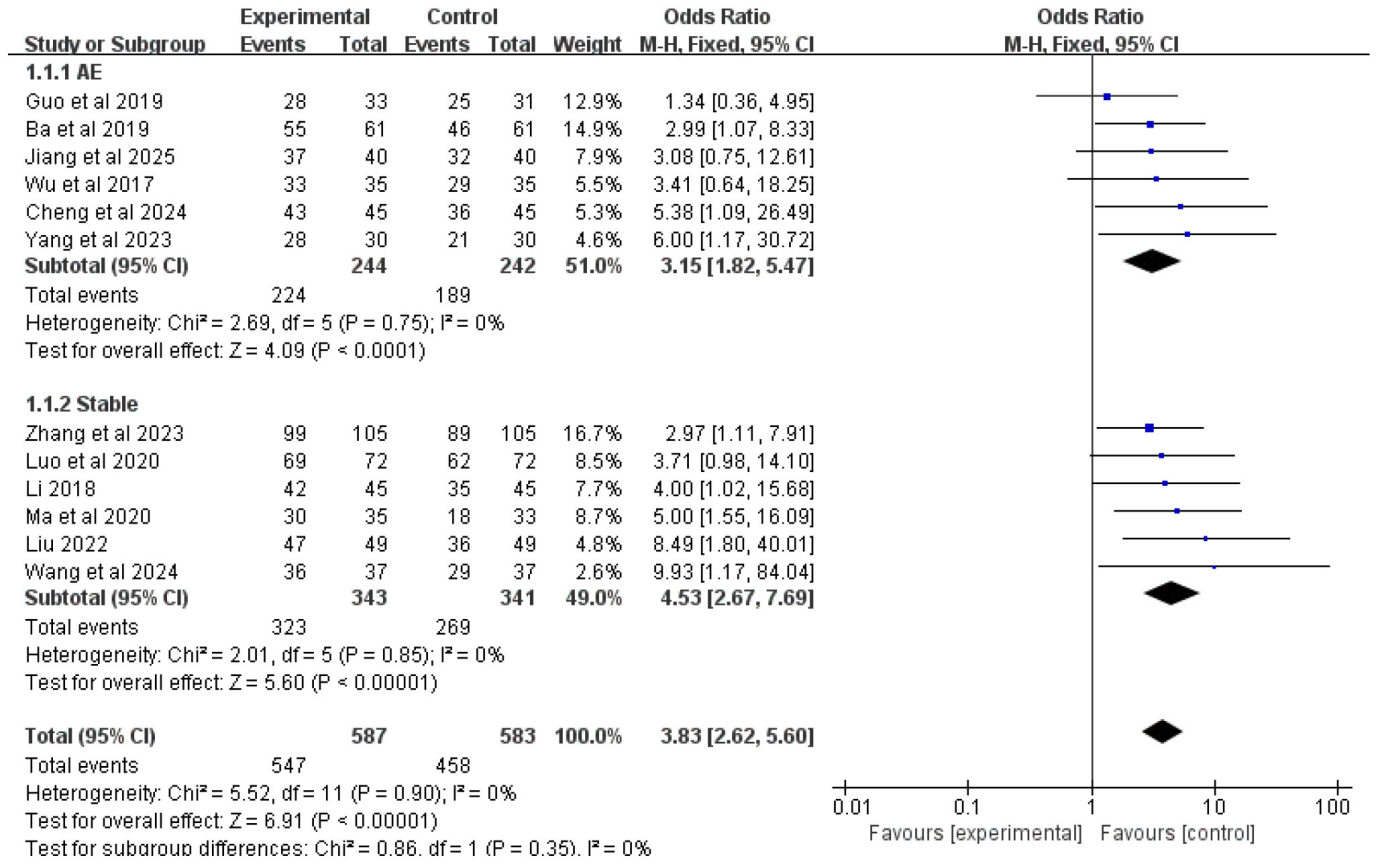
Forest plot of treatment effectiveness rate analysis.

Subgroup analysis by disease stage showed that for COPD patients with AMH during acute exacerbation, the combined therapy showed higher treatment effectiveness rate than Western medicine alone [OR = 3.15, 95% CI (1.82, 5.47), *P* < 0.0001]; for stable COPD patients with AMH, the integrated therapy also demonstrated superior treatment effectiveness [OR = 4.53, 95% CI (2.67, 7.69), *P* < 0.00001]. The complete results are presented in Figure 3.

#### 3.4.2 Forced vital capacity (FVC)

A total of six studies included FVC as an outcome measure. Given the substantial heterogeneity observed among these studies (*I*^2^ = 84%), a random-effects model was selected for analysis. The meta-analysis results demonstrated that the combination of Chinese herbal medicine (utilizing SSPR principles) with conventional Western medicine produced significantly greater improvements in FVC compared to Western medicine alone [MD = 0.32, 95% CI (0.16, 0.49), *P* < 0.0001].

**Figure 4:**
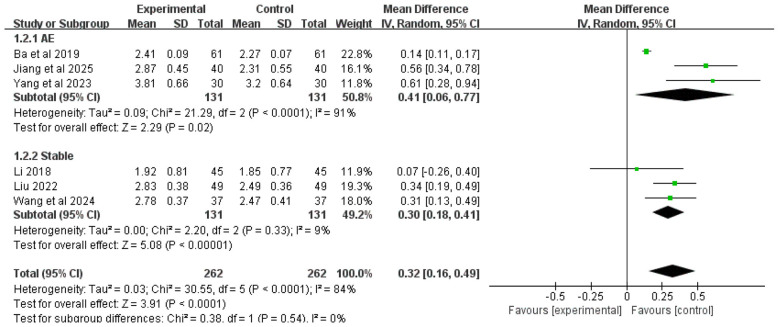
Forest plot of FVC analysis.

Subgroup analysis based on disease phase revealed distinct outcomes. For COPD patients experiencing acute exacerbation with AMH, the integrated therapy showed superior FVC improvement over Western medicine alone [MD = 0.41, 95% CI (0.06, 0.77), *P* = 0.02]. Similarly, for stable COPD patients with AMH, the combined treatment approach also yielded significantly better FVC outcomes [MD = 0.30, 95% CI (0.18, 0.41), *P* < 0.00001]. The complete results are presented in Figure 4.

#### 3.4.3 Absolute FEV_1_ value

Eight studies reported the absolute FEV_1_ value as an outcome measure. Due to significant heterogeneity among studies (*I*^2^ = 83%), a random-effects model was employed for analysis. The meta-analysis demonstrated that the addition of Chinese herbal medicine with SSPR effects to conventional Western medicine resulted in significantly greater improvement in absolute FEV_1_ values compared to Western medicine alone [MD = 0.25, 95% CI (0.23, 0.27), *P* < 0.00001].

**Figure 5:**
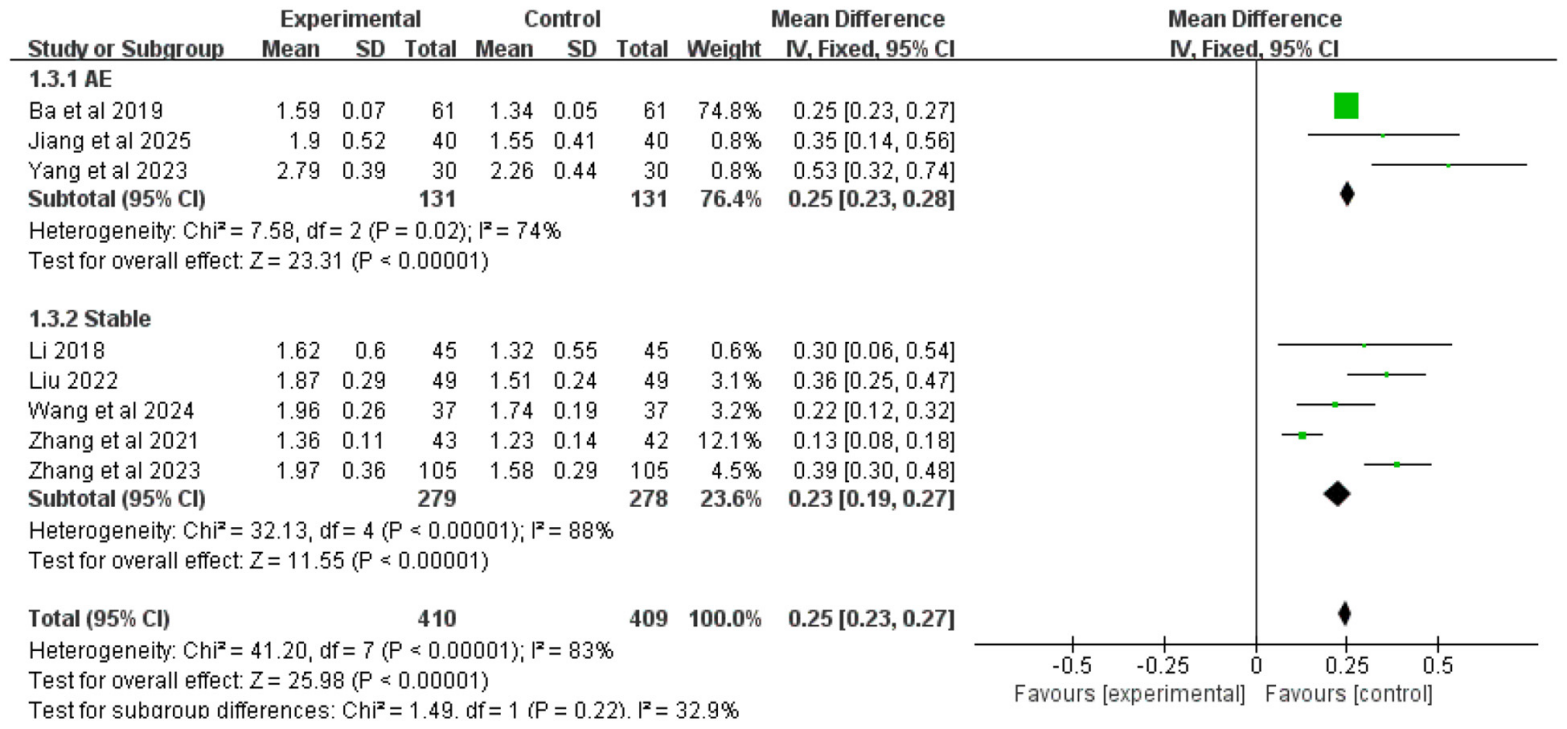
Forest plot of absolute FEV_1_ analysis.

Subgroup analysis by disease stage showed that for COPD patients with AMH during acute exacerbation, the combined therapy was more effective in improving absolute FEV_1_ values than Western medicine alone [MD = 0.25, 95% CI (0.23, 0.28), *P* < 0.00001]. Similarly, for stable COPD patients with AMH, the integrated treatment also showed superior efficacy in enhancing absolute FEV_1_ values [MD = 0.23, 95% CI (0.19, 0.27), *P* < 0.00001]. The complete results are presented in Figure 5.

#### 3.4.4 FEV_1_/FVC ratio

Seven studies included FEV_1_/FVC ratio as an outcome measure. Due to high heterogeneity among studies (*I*^2^ = 99%), a random-effects model was used for analysis. The results showed that overall, the addition of Chinese herbal medicine with SSPR effects to conventional Western medicine significantly improved FEV_1_/FVC ratio compared to Western medicine alone [MD = 5.14, 95% CI (0.39, 9.89), *P* = 0.03].

**Figure 6:**
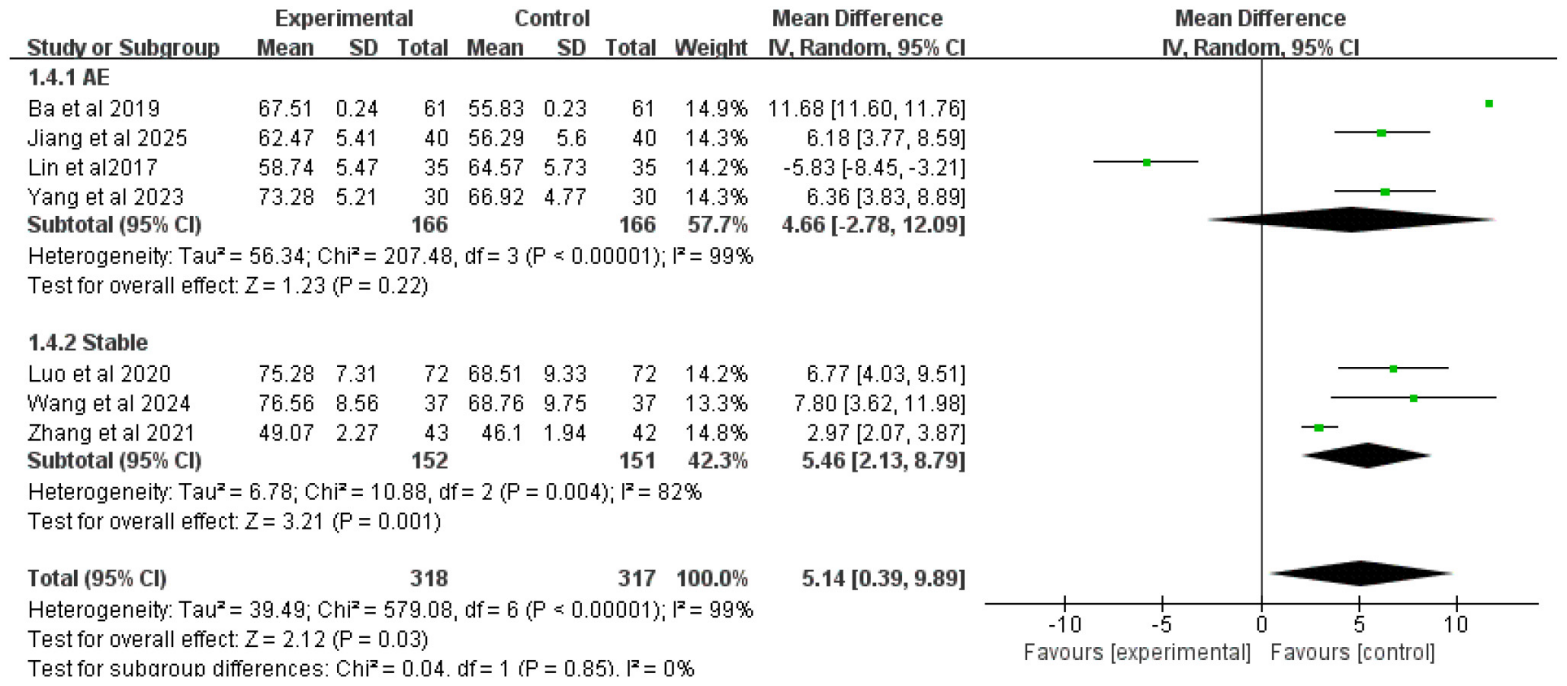
Forest plot of absolute FEV_1_/FVC ratio analysis.

Subgroup analysis by disease stage showed that for COPD patients with AMH during acute exacerbation, there was no statistically significant difference in FEV_1_/FVC ratio improvement between the combined therapy and Western medicine alone [MD = 4.66, 95% CI (−2.78, 12.09), *P* = 0.22]; for stable COPD patients with AMH, the integrated therapy demonstrated superior efficacy in improving FEV_1_/FVC ratio [MD = 5.46, 95% CI (2.13, 8.79), *P* = 0.001]. The complete results are presented in Figure 6.

#### 3.4.5 MUC5AC

Eleven studies included MUC5AC as an outcome measure. Due to high heterogeneity among studies (*I*^2^ = 97%), a random-effects model was used for analysis. The results showed that overall, the addition of Chinese herbal medicine with SSPR effects to conventional Western medicine significantly reduced MUC5AC levels compared to Western medicine alone [SMD = −1.16, 95% CI (−2.04, −0.27), *P* = 0.01].

**Figure 7:**
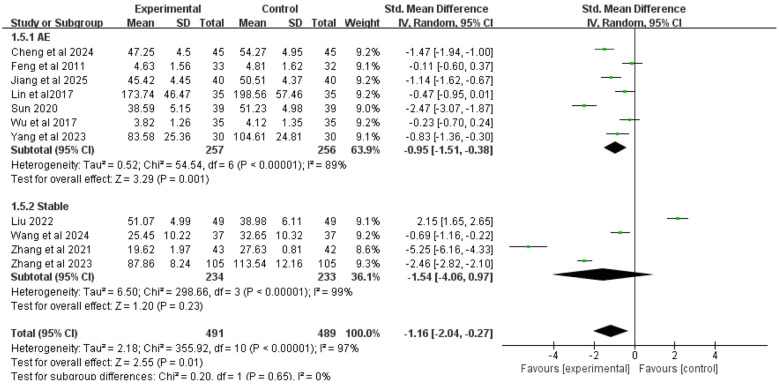
Forest plot of MUC5AC analysis.

Subgroup analysis by disease stage showed that for COPD patients with AMH during acute exacerbation, the combined therapy was more effective in reducing MUC5AC than Western medicine alone [SMD = −0.95, 95% CI (−1.51, −0.38), *P* = 0.001]; for stable COPD patients with AMH, there was no statistically significant difference in MUC5AC reduction between the integrated therapy and Western medicine alone [SMD = −1.54, 95% CI (−4.06, 0.97), *P* = 0.23]. The complete results are presented in Figure 7.

#### 3.4.6 TCM symptom score

Ten studies included TCM syndrome scores as an outcome measure. Due to significant heterogeneity among studies (*I*^2^ = 93%), a random-effects model was employed for analysis. The results demonstrated that, overall, the addition of Chinese herbal medicine with SSPR effects to conventional Western medicine significantly reduced TCM syndrome scores compared to Western medicine alone, with statistically significant differences [SMD = −1.53, 95%CI (−2.13, −0.94), *P* < 0.00001].

**Figure 8:**
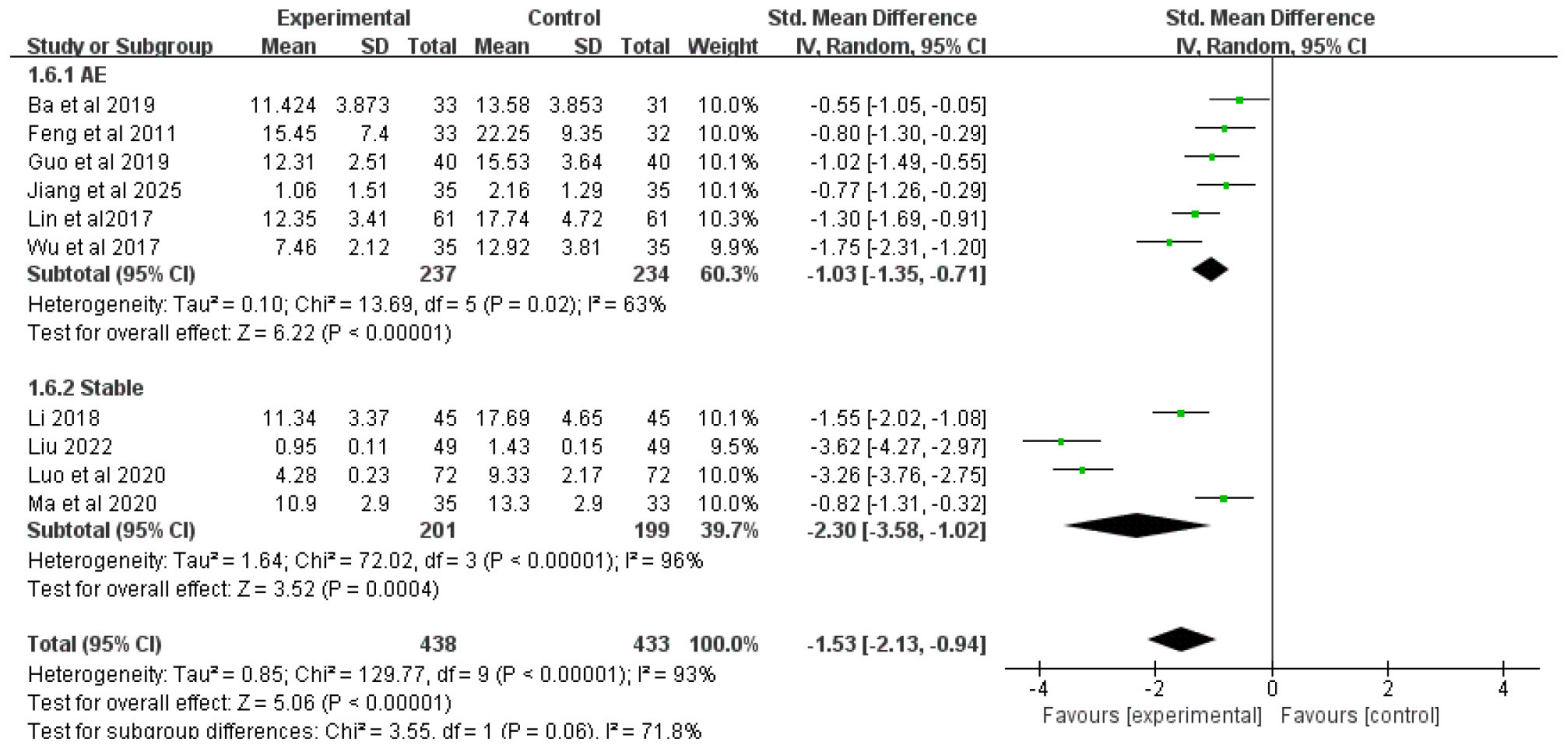
Forest plot of TCM symptom score analysis.

Subgroup analysis by disease stage showed that: For COPD patients with AMH during acute exacerbation, the combined therapy was more effective in reducing TCM syndrome scores than Western medicine alone [SMD = −1.03, 95% CI (−1.35, −0.71), *P* < 0.00001]; For stable COPD patients with AMH, the integrated approach also demonstrated superior efficacy in lowering TCM syndrome scores [SMD = −2.30, 95% CI (−3.58, −1.02), *P* = 0.0004]. The complete results are presented in Figure 8.

#### 3.4.7 IL-8

Five studies included IL-8 levels as an outcome measure. Due to significant heterogeneity among studies (*I*^2^ = 97%), a random-effects model was employed for analysis. The results demonstrated that, overall, the addition of Chinese herbal medicine with SSPR effects to conventional Western medicine significantly reduced IL-8 levels compared to Western medicine alone [SMD = −2.22, 95% CI (−2.48, −1.97), *P* < 0.00001].

**Figure 9:**
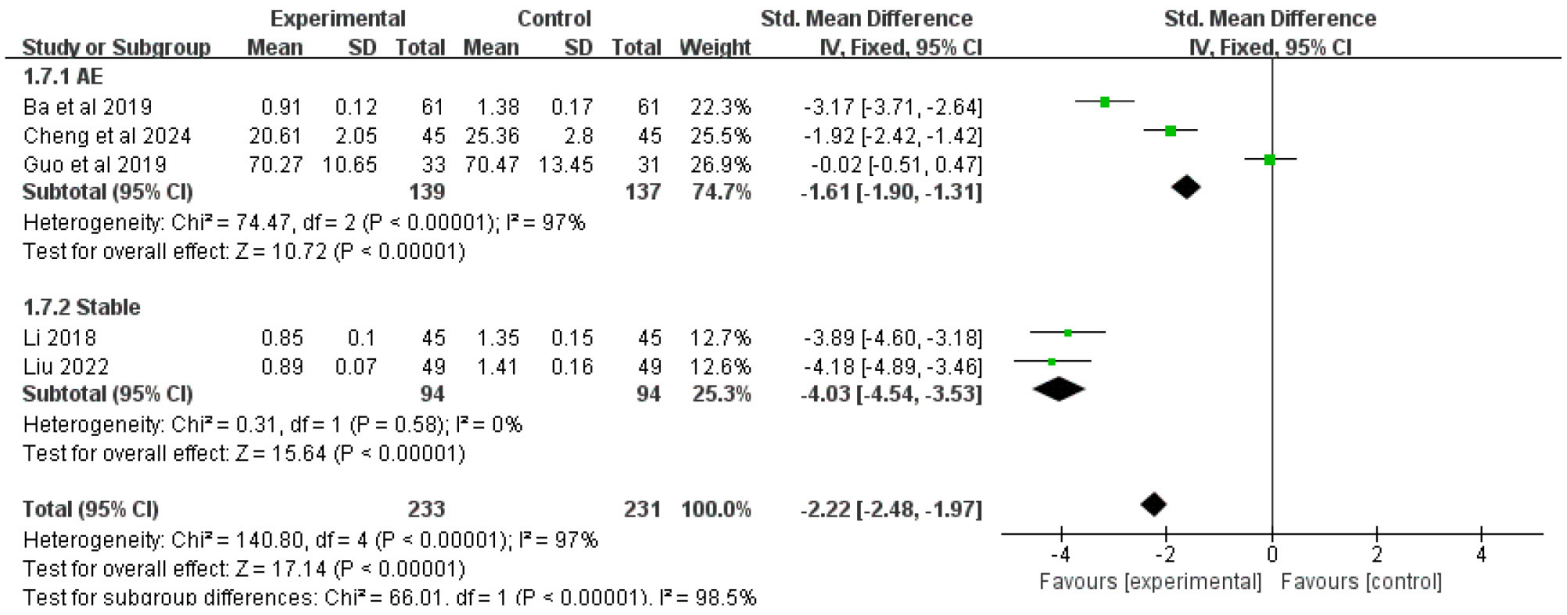
Forest plot of IL-8 analysis.

Subgroup analysis by disease stage showed that: For COPD patients with AMH during acute exacerbation, the combined therapy was more effective in reducing IL-8 levels than Western medicine alone [SMD = −1.61, 95% CI (−1.90, −1.31), *P* < 0.00001]; For stable COPD patients with AMH, the integrated approach also demonstrated superior efficacy in lowering IL-8 levels [SMD = −4.03, 95% CI (−4.54, −3.53), *P* < 0.00001]. The complete results are presented in Figure 9.

#### 3.4.8 Safety and adverse reactions

Five studies reported safety evaluations or adverse reactions. Among these, three studies explicitly stated no occurrence of adverse reactions during treatment. The remaining two studies reported adverse reactions such as coughing and nausea in both the treatment and control groups, with no statistically significant difference in incidence rates between groups. No severe adverse reactions were reported in any of the included studies, indicating that the SSPR therapy demonstrates good safety in treating COPD with AMH.

#### 3.4.9 Publication bias analysis

**Figure 10:**
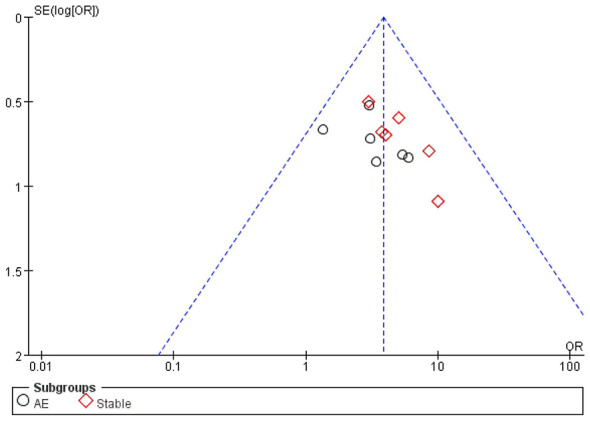
Funnel plot of treatment effectiveness rate.

Funnel plot analysis was conducted on studies reporting treatment effectiveness rate (the most frequently reported outcome). The results showed an approximately symmetrical distribution of studies (Figure 10), suggesting low likelihood of publication bias in the included literature. To further validate this conclusion, Egger's test yielded *P* = 0.075 (>0.05), confirming the low probability of publication bias in the studies incorporated in this meta-analysis.

## 4 Discussion

Treatment effectiveness rate provides the most direct comparison of therapeutic efficacy between regimens. FVC, absolute FEV_1_, and FEV_1_/FVC ratio are crucial for COPD diagnosis and pulmonary function assessment (1). MUC5AC, as a major mucus component (20), reflects the severity of pathological mucus hypersecretion in COPD patients. TCM syndrome scores quantify syndrome manifestations reflecting disease severity. IL-8, an inflammatory biomarker related to systemic inflammation in COPD (21), where lower levels indicate less severe pulmonary infection, facilitating mucus clearance and slowing disease progression (22).

Our findings demonstrate that for COPD patients with AMH in either acute exacerbation or stable phases, adding SSPR herbal medicine to conventional Western treatment significantly outperformed Western medicine alone in improving treatment response rates, FVC, absolute FEV_1_, reducing TCM syndrome scores, and lowering IL-8 levels–all with statistical significance. However, for acute exacerbation patients, the two approaches showed no statistically significant difference in improving FEV_1_/FVC ratio, suggesting the herbal treatment may be more suitable for improving pulmonary function in stable COPD patients. For stable patients, the two approaches showed no statistically significant difference in reducing MUC5AC, indicating the herbal treatment may be more effective for alleviating pathological mucus hypersecretion during acute exacerbations. All studies also indicated good safety of this approach.

The pharmacological mechanism behind the significant efficacy of SSPR herbal treatment for COPD mucus hypersecretion lies in the common formula components: *Poria* and *Atractylodis Macrocephalae Rhizoma* strengthen spleen and drain dampness, both resolving accumulated resolving accumulated fluids and cutting off phlegm production; *Citri Reticulatae Pericarpium, Pinelliae Rhizoma*, and *Magnoliae Officinalis Cortex* dry dampness, transform phlegm, regulate qi and harmonize the middle; *Platycodonis Radix* and *Peucedani Radix* diffuse and descend lung qi, relieve cough and dispel phlegm—embodying Zhu Danxi's principle that “in treating phlegm, directing qi downward is paramount.” Additional components may include: *Trichosanthis Pericarpium, Scutellariae Radix, Mori Cortex*, and *Fritillariae Thunbergii Bulbus* to clear heat and transform phlegm; *Salviae Miltiorrhizae Radix et Rhizoma, Chuanxiong Rhizoma*, and *Persicae Semen* quicken blood and transform stasis; *Cinnamomi Ramulus* and *Zingiberis Rhizoma* warm yang and transform fluids; *Ophiopogonis Radix* and *Schisandrae Chinensis Fructus* nourish yin and generate fluids; *Gecko* and *Schisandrae Chinensis Fructus* supplement kidney and grasp qi; finally *Glycyrrhizae Radix et Rhizoma* strengthens spleen and drains dampness, sweet-warm supplementation of qi, and harmonizes all formula components.

The formulas combine warming with moistening, treating both root and branch, warming yang to transform fluids and restore spleen transport, strengthening spleen to resolve phlegm and smooth qi movement, percolating dampness to prevent congestion.

In the acute exacerbation stage of COPD, the main pathogenesis is “dominance of pathogenic factors,” and treatment should focus on “eliminating pathogenic factors.” The main pathological characteristics often involve external pathogenic factors triggering internal phlegm, with pathological products such as phlegm, heat, and stasis blocking the airway, leading to airway obstruction and acute inflammation attacks. The most common TCM syndromes are “phlegm-heat congestion in the lung,” “phlegm-dampness obstruction in the lung,” and “external cold and internal fluid retention.” The formula combinations mostly extensively use drugs for resolving phlegm, clearing heat, dispelling exterior, and resolving stasis. The therapeutic effect results show that these traditional Chinese medicine compound formulas have significant effects in reducing mucin MUC5AC and key inflammatory factors IL-8. This precisely corresponds to its “clearing heat and resolving phlegm” treatment method, directly acting on the core pathological process of airway mucus hypersecretion and neutrophil inflammation in the acute exacerbation stage of COPD. In the drug combination structure for the acute exacerbation stage of COPD, the principal herbs are mostly powerful drugs for clearing heat and resolving phlegm or promoting lung ventilation and asthma relief, while the auxiliary herbs mostly include drugs for regulating and resolving phlegm. The aim is to quickly open the airway and eliminate pathological products, but these drugs have limited effect on chronic and long-term structural airway remodeling (the fundamental cause of the continuous decline in FEV_1_/FVC).

In the stable stage of COPD, the main pathogenesis is “deficiency of the root,” and treatment should focus on “strengthening the root and consolidating the foundation.” During this stage, external pathogenic factors have temporarily disappeared, but the deficiency of the lung, spleen, and kidney becomes the main contradiction. The most common TCM syndromes are “lung qi deficiency,” “spleen and lung qi deficiency,” and “lung-kidney qi deficiency.” The core drugs in the formula combination shift to tonifying qi, strengthening the spleen, warming the kidney, and regulating and resolving water-dampness, phlegm, and removing stasis. The therapeutic effect results show that these traditional Chinese medicine compound formulas have significant effects in improving FEV_1_/FVC. This indicates that based on the foundation of eliminating phlegm and resolving heat, combining with tonifying qi and strengthening the essence drugs, can more effectively improve long-term airflow limitation and decline in lung function. This is precisely the embodiment of “strengthening the root and consolidating the foundation.” In the drug combination structure for the stable stage of COPD, the principal herbs change to tonifying qi and strengthening the foundation or warming the kidney and regulating breathing, and the auxiliary herbs mostly include herbs for tonifying the spleen, resolving phlegm, and promoting blood circulation. The aim is to restore the functions of the organs and eliminate the root cause. Such tonifying-based formulas may not be as direct and rapid as the “clearing heat and resolving phlegm” formulas used in the acute exacerbation stage in directly suppressing mucus secretion (MUC5AC), but they are more focused on regulating the environment as a whole, reducing the “root” of phlegm production (spleen deficiency leads to phlegm), rather than directly “eliminating” the existing large amounts of phlegm markers.

## 5 Conclusions

In summary, our study systematically screened randomized controlled trials since 2010 on SSPR herbal treatments for COPD mucus hypersecretion, ultimately including 16 studies involving 1,468 COPD patients. From an evidence-based medicine perspective, we systematically evaluated and confirmed that adding this approach to conventional Western treatment yields better clinical efficacy than Western medicine alone for COPD mucus hypersecretion, with good safety.

For patients with low lung function in the acute exacerbation stage, although treatment focuses on “eliminating pathogenic factors” as a priority, based on the theory that “deficiency of the lung, spleen, and kidney constitutes the root of the disease,” in the formula combination, it is necessary to consider timely and moderate addition of tonifying and stabilizing foundation drugs. This can not only prevent severe attacks that damage the body's essence, but also help the body resist pathogenic factors and transition smoothly to the stable stage “tonifying the root and stabilizing the foundation” treatment after controlling acute symptoms, reflecting a phased strategy of “simultaneous attack and supplementation.” For patients in the stable stage still having prominent mucus hypersecretion as the main problem, it is possible to consider adding commonly used clearing heat and resolving phlegm drugs in the tonifying and stabilizing foundation formula, forming a dynamic combination to balance “symptom” and “root.”

Study limitations include most included studies were small-sample trials providing low-quality evidence; high heterogeneity between studies may relate to treatment duration and case characteristics; while all were RCTs, implementation carried some selection bias risk requiring cautious interpretation. Larger-sample, multicenter, high-quality RCTs are needed to guide clinical application and draw more reliable conclusions, further verifying efficacy and safety.
